# Complete-Genome Analysis of Echovirus-30 Isolated from an Encephalitis Case in India Revealed Distinct Mutations

**DOI:** 10.3390/microorganisms13071580

**Published:** 2025-07-04

**Authors:** Rishabh Waghchaure, Jithin Kunjumon, Alfia Fathima Ashraf, Ranjana Mariyam Raju, Anita Shete, Sarah Cherian, Mallika Lavania

**Affiliations:** 1 Enteric Viruses Group, ICMR-National Institute of Virology, 20-A, Dr. Ambedkar Road, Pune 411001, India; rishabhw1997@gmail.com (R.W.); jithinalpy97@gmail.com (J.K.); 2 Bioinformatics Group, ICMR-National Institute of Virology, 20-A, Dr. Ambedkar Road, Pune 411001, India; alfiyafathimaasharaf@gmail.com (A.F.A.); rmariyamr42@gmail.com (R.M.R.); 3 Maximum Containment Laboratory, ICMR-National Institute of Virology, Sus Road, Pashan, Pune 411021, India; anitaaich2008@gmail.com; 4 Academy of Scientific and Innovative Research (AcSIR), Ghaziabad 201002, India

**Keywords:** Echovirus 30, whole-genome sequencing, acute encephalitis

## Abstract

Echovirus 30 (E-30), a member of the Enterovirus B species, is frequently linked to neurological illnesses such as aseptic meningitis, encephalitis, and hand, foot, and mouth disease. In this study, we present the complete-genome analysis of an Echovirus 30 strain isolated from cerebrospinal fluid (CSF) and stool samples of a pediatric encephalitis case in Kerala, India, during 2023. A comparative genomic investigation was carried out using a dataset of 111 human E-30 isolates, encompassing 116,991 mutation records. This analysis revealed six distinct non-synonymous amino acid substitutions uniquely present in the isolate PQ472410.1, which may be associated with pathogenicity and/or neurotropic behavior. To the best of our knowledge, this represents the first complete-genome sequence report of E-30 from an encephalitis case in India. These findings contribute valuable information to the understanding of E-30’s molecular epidemiology and evolution and offer vital data for enhancing surveillance and response strategies against enteroviral infections.

## 1. Introduction

Enteroviruses are part of the Enterovirus genus within the Picornaviridae family. The major enterovirus groups associated with human illnesses include EV-A, EV-B, EV-C, and EV-D, with E-30 falling under the EV-B group. E-30 has been extensively studied and documented on a global scale, showing strong links to various infectious conditions such as encephalitis, myocarditis, and hand, foot, and mouth disease. Numerous retrospective studies analyzing clinical samples such as feces, serum, and cerebrospinal fluid from various countries have consistently identified E-30 as a leading cause of aseptic meningitis outbreaks in Asia, Europe, and the Americas in recent times [1,2,3,4].

Enteroviruses, particularly Echovirus 30 (E-30), are well known for their recurrent outbreaks, which pose significant public health challenges [5,6,7,8]. E-30 is a non-enveloped virus with a single-stranded, positive-sense RNA genome of approximately 7.4 kilobases. The genome comprises a single open reading frame (ORF) flanked by 5′ and 3′ untranslated regions (UTRs). This ORF encodes a large polyprotein, which is subsequently cleaved into structural proteins (VP1–VP4) and non-structural proteins. Among these, the VP1 protein is of particular importance for molecular epidemiology, as it contains critical neutralization epitopes and serves as the primary region used for the serotyping and genotyping of enteroviruses [9,10,11].

E-30 outbreaks tend to occur in cyclical patterns, typically peaking every 3 to 5 years, especially in temperate regions [12]. These periodic surges are believed to result from a combination of declining population immunity, viral evolution through mutation and recombination, and favorable environmental conditions that enhance transmission. In China, surveillance studies have identified E-30, alongside EV-A71 and CV-B5, as major contributors to viral meningitis and hand, foot, and mouth disease (HFMD) in children. A notable outbreak in 2014 in Shandong Province saw numerous children presenting with clinical features typical of viral meningitis, such as fever, vomiting, photophobia, and neck stiffness, raising significant health concerns [9].

E-30 continues to be a pressing concern in Europe as well. In 2018, the European Centre for Disease Prevention and Control (ECDC) reported a marked increase in E-30 cases across multiple countries, including Denmark, Germany, and the Netherlands. This prompted detailed molecular investigations, which revealed that many of the circulating strains belonged to newly emerging genogroup VI lineages, indicating active viral evolution and the emergence of novel variants [5]. These findings underscore the importance of continuous molecular surveillance to detect and respond to evolving strains that may lead to widespread outbreaks.

Despite its clinical significance, there is currently no targeted antiviral therapy or approved vaccine for E-30. As a result, clinical management remains largely supportive, focusing on symptom relief and preventing complications. Given the virus’s ability to cause CNS infections and its unpredictable outbreak behavior, comprehensive molecular characterization including whole-genome sequencing and phylogenetic analysis is essential. These approaches help clarify transmission dynamics, identify potential recombination events, and detect genetic markers associated with virulence and neurotropism. Moreover, recombination is widely recognized as a major driver of enteroviral genetic diversity, allowing viruses to adapt to new hosts and environmental conditions [11,13].

Against this backdrop, our study provides important insights through the isolation and analysis of an E-30 strain from the cerebrospinal fluid (CSF) of a child diagnosed with aseptic meningitis. By conducting whole-genome sequencing and comparative phylogenetic analysis, we aimed to better understand the virus’s genetic makeup, its evolutionary context, and possible recombination patterns. These findings are crucial not only for advancing our understanding of E-30 pathogenesis but also for informing clinical management and strengthening public health surveillance and response strategies in affected regions.

As a designated reference laboratory for viral diagnostics, our department received samples from a two-year-old child in Kerala, India. The child had been experiencing fever for five days along with episodes of seizures. Symptoms included lethargy, irritability, and abnormal movements. Based on clinical assessment and laboratory findings, the case was diagnosed as viral encephalitis. Prior to the illness, the child was healthy, fully vaccinated, and had achieved all age-appropriate developmental milestones.

## 2. Materials and Methods

The clinical specimens including oropharyngeal/nasopharyngeal swabs, CSF, stool and serum, were referred to the laboratory in cold chain to rule out the viral etiology of acute encephalitis syndrome in the patient.

### 2.1. Ethical Clearance

This study was reviewed and approved by the Ethics Committee of the ICMR-National Institute of Virology, Pune (MP-24A-7N), in accordance with established ethical guidelines for biomedical research involving human samples.

### 2.2. Virus Isolation

The isolation of the virus was performed in the rhabdomyosarcoma (RD) cell line following WHO protocols [14,15,16,17,18] for virus characterization. RD cells are large, multinucleated, spindle-shaped cells originally isolated from the muscle tissue of a 7-year-old female patient with pelvic rhabdomyosarcoma refractory to cyclophosphamide and radiation therapy. The RD cell line (ATCC CCL-136) was obtained from the American Type Culture Collection and maintained under standard culture conditions as previously described [18]. RNA was extracted from the cell culture supernatant using a Qiagen kit and tested by qRT-PCR to confirm the presence of enterovirus. The sample tested positive for pan-enterovirus by qRT-PCR with a Ct value of 25. Genotyping was confirmed using semi-nested RT-PCR targeting the VP1 region [19]. Sequence identity was determined through a Nucleotide BLAST search (https://blast.ncbi.nlm.nih.gov/blast/Blast.cgi?PROGRAM=blastn&PAGE_TYPE=BlastSearch&LINK_LOC=blasthome accessed on 21 April 2025).

### 2.3. Reverse Transcription Polymerase Chain Reaction (RT-PCR), Sequencing, and Typing

RT-PCR, sequencing, and typing were performed following a previously described protocol [20]. Viral RNA was extracted from the supernatants of infected cells using the Body Fluid Viral DNA/RNA Miniprep Kit (Axygen, Union City, CA, USA). RT-PCR was conducted with the PrimeScript One Step RT-PCR Kit Ver.2 (DSS Takara Bio India Pvt. Ltd., New Delhi, India).

To amplify partial VP1 sequences, the primers AN89 and AN88 were used [20]. Complete-genome fragments were amplified and sequenced using multiple primer pairs, as summarized in Additional File 1 (Table S1), on an ABI PRISM 3100 Genetic Analyzer (Applied Biosystems, Waltham, MA, USA). The positive amplification products were sequenced by Tsingke Biological Technology Co., Ltd. (Kunming, China). Enterovirus classification was performed using the Enterovirus Genotyping Tool Version 2.17.

VP1-encoding sequences and complete genomes were compared with publicly available sequences in GenBank using BLAST (https://blast.ncbi.nlm.nih.gov/Blast.cgi?PROGRAM=blastn&PAGE_TYPE=BlastSearch&LINK_LOC=blasthome assessed on 21 April 2025) [21]. The assembly parameters were set at the default values. The algorithm that was best suited based on the sample size was chosen using the auto command.

### 2.4. Whole Genome Assembly and Phylogeny

The whole genome of the isolate was sequenced using the random primer strategy, which utilized the TruSeq Stranded mRNA LT Library Preparation Kit on the Illumina Miniseq platform. For the whole-genome sequencing (WGS) of the positive-sense single-stranded RNA virus Echovirus 30, the TruSeq Stranded mRNA LT Library Preparation Kit (Illumina) was adapted accordingly. Total RNA extracted from the supernatant of virus-infected cell cultures was subjected to random fragmentation, followed by reverse transcription using random primers to achieve comprehensive genome coverage. Strand specificity was maintained by incorporating dUTP during second-strand synthesis, allowing the selective degradation of the second strand. The resulting double-stranded cDNA was then end-repaired, adenylated at the 3′ ends, and ligated to indexed sequencing adapters. After library construction, PCR amplification was performed, and the libraries were subsequently quantified and evaluated for quality before being sequenced on an Illumina platform. After RNA library preparation and normalization, the library was loaded onto the platform, and FASTQ data were analyzed with CLC Genomics Workbench version 20, employing de novo assembly and reference mapping.

For the molecular epidemiology study, we retrieved VP1 and 110 whole-genome sequences from GenBank and selected 52 representative strains for genotyping. The aligned sequences were then subjected to phylogenetic reconstruction using the maximum likelihood (ML) approach, implemented via IQ-TREE v2.2.0. The best-fit substitution model was automatically selected by IQ-TREE based on the Bayesian information criterion (BIC). Tree robustness was assessed using 1000 ultrafast bootstrap replicates. All parameters were kept at their default settings unless specified. The final tree was visualized and annotated using Interactive Tree of Life (iTOL version 6).

Phylogenetic analysis of VP1 was conducted using MEGA software version 7, calculating genetic distances using the Kimura two-parameter model [22,23]. The full genome sequence of the Indian isolate (passage 4) of E-30 has been deposited in GenBank under the accession number PQ472410. An initial phylogenetic tree based on neighbor joining (NJ) was constructed based on the VP1 gene to verify the serotype and lineage assignment of the isolate. Given the high resolution required for evolutionary clustering, a subsequent maximum likelihood phylogeny using full genome sequences (*n* = 110) was conducted to refine the placement of PQ472410.1 within the global Echovirus 30 landscape. The whole-genome IQ-TREE phylogenetic tree forms the basis of all final analyses, interpretations, and conclusions presented in this study.

### 2.5. Mutation Analysis Based on Whole-Genome Sequencing

A comprehensive mutation analysis at the amino acid level was performed on all available whole-genome sequences from 2005 to 2019, using the prototype strain AF162711 (Bastianni strain) as a reference. Nucleotide sequences were aligned with the prototype strain using MAFFT v7.5266 [24] to identify nucleotide substitutions. The aligned FASTA file was then analyzed using an in-house Python v3.10.12 pipeline to detect mutations across the polypeptide regions in a tabular format. To validate the identified mutations, we employed MEGA11, where the aligned FASTA file was translated to assess specific amino acid changes.

## 3. Results

### 3.1. Isolation and Molecular Characterization of Virus

**Figure 1:**
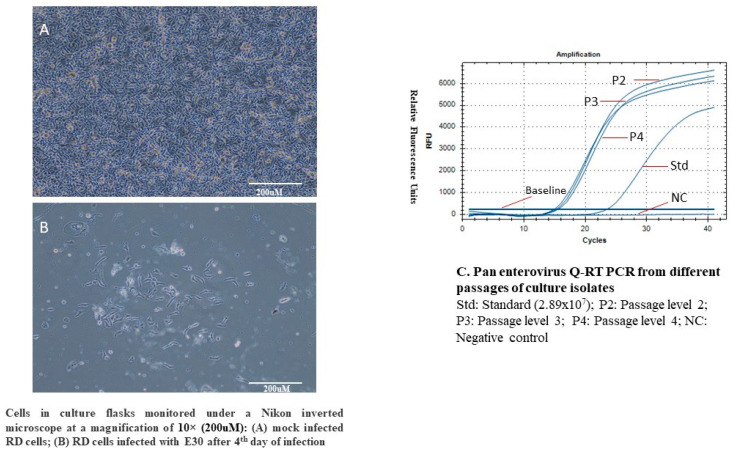
CPEs appeared after infection of RD cells with E-30. (**A**) Mock-infected RD cells. (**B**) RD cells infected with E-30 after 4th day of infection. (**C**) Pan-enter q-RT PCR from different passages of culture isolates.

Virus isolation and characterization were carried out using the RD (rhabdomyosarcoma) cell line, which was cultured under standard laboratory conditions. The cells were inoculated and monitored over four successive blind passages (P1 to P4), performed in duplicate. Consistent viral replication was observed throughout the passages, with marked cytopathic effects (CPEs) appearing from day 4 post-inoculation (Figure 1A,B).

The observed CPEs were typical of enteroviral infection and included cellular rounding, aggregation into dense clumps, increased cytoplasmic granularity suggestive of inclusion body formation, and detachment from the culture substrate. The extent of cellular damage intensified with each passage, and monolayers showing over 90–95% CPEs were selected for downstream analysis.

Viral lysates from passage 4 (P4) cultures demonstrating maximum CPEs were harvested for molecular confirmation. RT-qPCR targeting the conserved pan-enterovirus region confirmed the presence of enteroviral RNA in the samples (Figure 1C). Further verification using VP1 gene-specific RT-PCR also yielded a positive result, indicating the successful amplification of the VP1 region, which is critical for enterovirus identification and serotyping.

These findings confirm that the virus was successfully propagated in RD cells and reliably identified as an enterovirus. The reproducibility of CPEs, coupled with the molecular detection of viral RNA in both pan-enterovirus and VP1-specific assays, strongly supports the authenticity and integrity of the viral isolate.

### 3.2. Whole-Genome Sequencing and Phylogenetic Analysis

**Figure 2:**
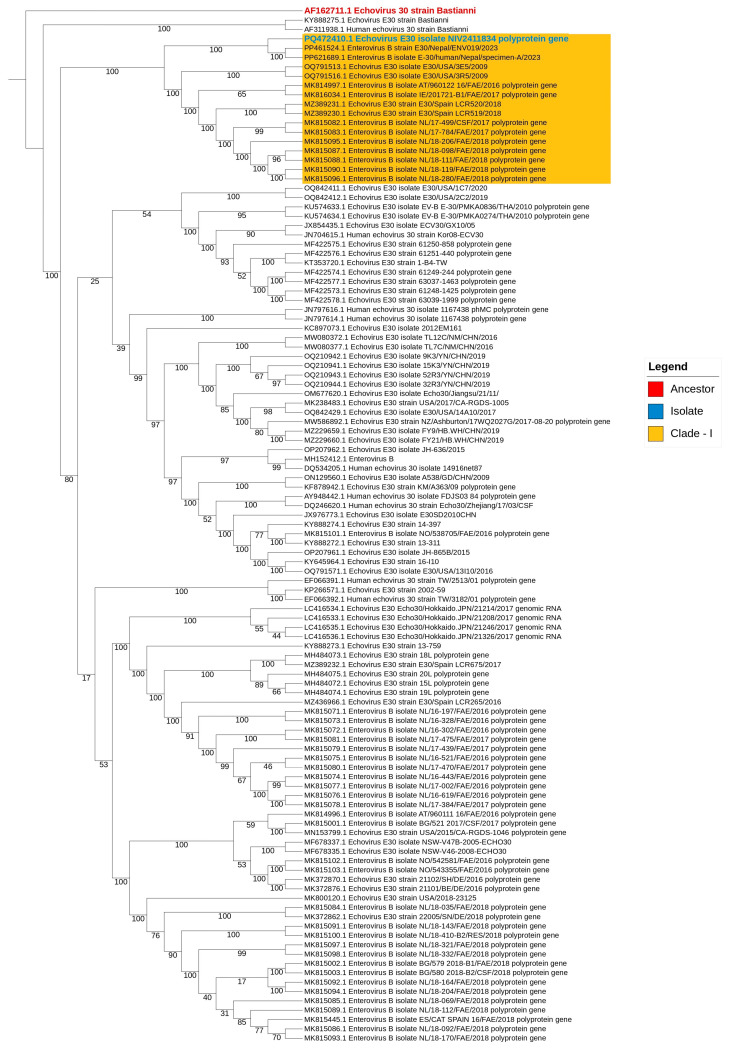
The full-genome maximum likelihood phylogenetic tree shows the clustering of the Indian isolate (blue) within Clade-I (orange), alongside closely related global strains. The reference strain is marked in red. Bootstrap values indicate branch support (1000 replicates).

The further positive passages were further investigated for full-genome sequencing. Full-genome sequencing was performed and phylogenetic analyses of this isolate revealed a genotype distributed to E-30 (Enterovirus B) [Figure 2]. The evolutionary history was inferred using the maximum likelihood (ML) method. The whole-genome sequences of the isolate (four different passages) isolated in India in 2023 were determined. These sequences ranged from 7403 to 7426 nucleotides in length and contained an open reading frame (ORF) of 6585 nucleotides, encoding a polyprotein of 2194 amino acids. The ORF was flanked by a noncoding 5′-UTR of 739–754 nucleotides and a noncoding 3′-UTR of 91–113 nucleotides. The whole-genome sequences showed 99.4–99.7% identity at the nucleotide level.

A comprehensive phylogenetic analysis was carried out for the E-30 isolate PQ472410.1 (passage 4) to explore its evolutionary relationships and genetic proximity to globally circulating Echovirus 30 strains. The analysis utilized the complete VP1 coding sequence of the isolate, comparing it with 110 globally representative E-30 sequences across diverse geographical regions and timeframes, with a focus on identifying unique amino acid variations and lineage clustering.

The phylogenetic tree was constructed using the maximum likelihood (ML) method in IQ-TREE, which revealed the clear stratification of global E-30 strains into multiple well-supported clades. The Indian isolate PQ472410.1 was positioned within Clade I, a strongly supported cluster that included 2023 isolates from Nepal (PP621689.1, PP461524.1) and strains from the USA (OQ791513.1, OQ791516.1), Spain (MZ389231.1, MZ389230.1), and the Netherlands (MK815082.1, MK815083.1, MK815095.1, MK815087.1, MK815088.1, MK815090.1, MK815096.1). This grouping received robust bootstrap support exceeding 90%, confirming a high degree of phylogenetic reliability.

Of particular interest is that the Indian isolate showed the greatest genetic similarity to a Nepalese strain from 2023, supported by a bootstrap value of 100%. This close relationship suggests a recent common ancestor and points to either shared transmission pathways or concurrent regional evolution in the South Asian context. The extended internal branch leading to the Indian isolate indicates rapid divergence, potentially driven by local selective pressures or undetected circulation in the region prior to detection.

The prototype E-30 strain “Bastianni” occupied a basal position in the tree, serving as an ancestral reference point. This further supports the recent diversification of the Nepal–India sublineage and emphasizes its evolutionary distinction from earlier European (2016–2018), Chinese (2016–2019), and North American (2009–2017) E-30 clusters.

These findings point to the emergence of a genetically distinct and locally evolving E-30 lineage in South Asia. The close clustering of recent South Asian and European isolates underscores the dynamic nature of E-30 circulation and the potential for international spread. Importantly, this analysis highlights the critical need for sustained molecular surveillance and genomic monitoring in the region, especially in areas with limited reporting of enterovirus-related CNS infections. The early detection and characterization of emerging lineages are essential for timely outbreak response and informing public health interventions.

### 3.3. Mutation Analysis Based on Whole Genome Sequencing

Mutation analysis with the complete-genome sequences was performed against the prototype “Bastianni” strain AF162711.1. The comparative genomic investigation based on the dataset of 111 human E-30 isolates encompassed a total of 116,991 mutation records. Further, the mutation analysis revealed a total of six non-synonymous mutations across various genomic regions of the E-30 Indian isolate (Table 1), highlighting the ongoing evolution and adaptation of the virus. We attempted to correlate the six non-synonymous substitutions to possible effects on protein structure or function.

Strikingly, the RNA-dependent RNA polymerase (3D) accounts for 50% of these changes, followed by the major capsid protein (VP1-33.33%) and the protease (2A-16.66%). VP1-V43I and VP1-P258L occur in the capsid protein VP1, which plays a key role in capsid stability. In the VP1 capsid protein, the V43I mutation lies within the N-terminal region but outside the canonical BC loop (residues 80–90) implicated in receptor binding [25]. Although not directly within a known antigenic loop, the N-terminal extensions and C-terminal arms of capsid proteins play essential roles in maintaining capsid stability [26]. The P258L mutation, located near the C-terminus of VP1, may similarly affect capsid assembly and viral infectivity, as surface-exposed loops and terminal regions are known to influence receptor attachment and structural integrity. The T60A mutation in the 2A protease lies outside the catalytic triad but may affect enzymatic function, as structurally analogous residues such as V59 and H68 have been shown to be critical for interactions with host factors like SETD3 and for viral replication [25]. Three substitutions were detected in the 3D RNA-dependent RNA polymerase: K22R, H260L, and E431A. The K22R mutation falls within the N-terminal region (residues 1–30), which stabilizes the palm domain and is essential for overall polymerase architecture [27]. The H260L mutation is located in the palm domain, which houses catalytic motifs A–E and governs RNA synthesis. The E431A mutation lies within the thumb domain, which maintains interactions with the template–primer duplex during RNA replication [28].

## 4. Discussion

Enteroviruses are a diverse group of viruses, known to cause a wide array of clinical manifestations, ranging from mild conditions like hand, foot, and mouth disease to more severe diseases such as aseptic meningitis, encephalitis, paralysis, neonatal sepsis-like disease, myocarditis, respiratory infections, and acute hemorrhagic conjunctivitis. Despite their significant impact on public health, the full extent of enterovirus distribution and the associated disease burden remain poorly understood, particularly in regions like Europe and India. This is partly due to inconsistent surveillance systems across different countries, leading to gaps in the understanding of enterovirus epidemiology and the potential risks they pose.

Aseptic meningitis, one of the most severe manifestations of enterovirus infections, is commonly associated with E-30. This virus has been implicated in numerous outbreaks worldwide, especially in Europe and Asia, where it has been a significant cause of viral meningitis. Studies from European countries, notably in 2018, reported an uptick in E30 infections, suggesting the increased circulation of this virus. The higher positivity rates of E-30 detected in enteroviral samples from multiple nations compared to those in previous years highlighted the virus’s growing prevalence and its potential to cause large-scale outbreaks [29]. In India, however, systematic surveillance of enterovirus infections remains insufficient, making it difficult to assess the true burden of these infections. A study performed by Mann et al. [30] based on nonpolio enterovirus associated with nonpolio-acute flaccid paralysis in Northern India, identified different clusters co-circulating in India.

The last significant outbreak of E-30 in India occurred in 2004, during which a rise in viral meningitis cases was noted, and E-30 was identified as the primary causative agent [31]. Despite these occurrences, the lack of continuous surveillance means that enteroviral infections, including E-30 infections, remain largely underreported. This study presents the first full-genome sequence of E-30 from India, which belongs to Clade I, a strain that is also circulating in Europe (Spain and the Netherlands) and Nepal. This finding is significant as it not only provides genetic insights into the local strain but also contributes to global surveillance efforts aimed at tracking the movement and mutation of enteroviruses.

The whole-genome sequencing of the Echovirus 30 (E-30) strain isolated from a child with encephalitis in Kerala revealed six unique non-synonymous amino acid substitutions not observed in other global isolates. These mutations were located within three functionally crucial viral proteins: the RNA-dependent RNA polymerase (3D), the protease (2A), and the major capsid protein (VP1). Each of these proteins plays a pivotal role in the viral life cycle. 3D is essential for viral RNA synthesis [32], 2A facilitates polyprotein cleavage and the disruption of host cell translation [33], and VP1 mediates receptor binding and immune recognition [34].

Functional annotations and structural modeling suggest that these mutations could influence viral pathogenicity, particularly neurovirulence. Changes in VP1 may affect viral entry and host immune escape, while alterations in 3D and 2A could modulate replication dynamics and host interaction. The exclusive presence of these mutations in a case of encephalitis rather than the more common clinical presentation of aseptic meningitis raises the possibility of a genetic basis for CNS invasion. Although the precise role of these mutations requires confirmation through laboratory models, their potential involvement in enhancing neurotropism is noteworthy.

These findings identify candidate molecular markers that may be linked to virulence, providing a basis for future research into antiviral targets or diagnostic tools. The identification of novel mutations also exposes a broader issue: the lack of comprehensive genomic surveillance of enteroviruses in India and many other resource-limited settings. This genome sequence contributes significantly to the limited database of Indian E-30 isolates and offers a regional perspective on viral diversity and evolution. Given the periodic nature and international spread of enterovirus outbreaks, the inclusion of such data is critical to global surveillance efforts.

In an era of increased global mobility and climate-driven shifts in disease patterns, the real-time tracking of viral evolution is essential. This study adds to the molecular epidemiology of E-30 in South Asia and emphasizes the importance of early detection systems capable of identifying genetic markers linked to severe disease outcomes.

Furthermore, the findings underscore the urgency of strengthening molecular surveillance infrastructure in countries with historically limited diagnostic capacity. Integrating genomic tools such as next-generation sequencing and mutation analysis into routine public health practice can enhance outbreak preparedness and response. The timely identification of emerging variants could enable the rapid implementation of containment strategies, clinical triage protocols, and risk communication.

In conclusion, this work provides a detailed view of a potentially neurovirulent E-30 strain, serving as a bridge between clinical virology, molecular epidemiology, and public health action. It reinforces the essential role of genomic surveillance in recognizing and managing infectious threats in an interconnected world.

## 5. Conclusions

Comprehensive mutational and phylogenetic analyses revealed that the Echovirus 30 (E-30) isolate associated with the 2023 outbreak belongs to a genetically distinct subclade linked to Nepal. This variant is characterized by a cluster of non-synonymous mutations concentrated in essential viral proteins, notably the RNA-dependent RNA polymerase (3Dpol) and the capsid protein VP1. Among these, six amino acid substitutions appear to be unique to this strain, indicating possible adaptive evolution that could affect viral replication, immune escape mechanisms, and tissue specificity. The presence of these mutations suggests a distinct evolutionary pathway, potentially shaped by localized environmental or host-related selective pressures. These molecular changes may influence the virus’s antigenic profile and raise concerns regarding cross-protection by existing vaccines. Overall, the findings underscore the importance of sustained genomic monitoring and functional studies to assess the biological implications of emerging variants. Such efforts are essential for predicting outbreak potential, informing the development of diagnostics and therapeutics, and guiding public health strategies tailored to regional viral evolution.

## Figures and Tables

**Table 1:** Unique non-synonymous substitutions in our isolate (PQ472410.1).

Protein	Genomic Coordinate	Nucleotide Substitution	Amino Acid Position	Amino Acid Change
VP1	1831	gta → ata	43	V → I
VP1	2476	cca → cta	258	P → L
2A	2758	aca → gcg	60	T → A
3D	5260	aag → aga	22	K → R
3D	5974	cat → ctc	260	H → L
3D	6487	gaa → gca	431	E → A

## Data Availability

The genome sequence assembled in this study with annotation is deposited in the NCBI GenBank under accession number PQ472410. The authors confirm all supporting data, code, and protocols provided within the article.

## Supplementary Materials

The following supporting information can be downloaded at https://www.mdpi.com/article/10.3390/microorganisms13071580/s1: Table S1: Primers used for VP1 and VP2 gene specific RT-PCR and sequencing.

## Abbreviations

The following abbreviations are used in this manuscript:

E	Echovirus
EV	Enterovirus
CSF	Cerebrospinal Fluid
MMR	Measles Mumps Rubella
HBV	Hepatitis B Virus
