# Supplementary material for: Complete-Genome Analysis of Echovirus-30 Isolated from an Encephalitis Case in India Revealed Distinct Mutations
Source: Microorganisms. 2025 Jul 4;13(7):1580. doi: 10.3390/microorganisms13071580 (PMC12300617; doi:10.3390/microorganisms13071580)
Supplement: Supplementary file 1 [file microorganisms-13-01580-s001.zip › microorganisms-3686563-supplementary.pdf]

Table S1: Primers used for VP1 and VP2 gene specific RT-PCR and sequencing.

**Primers**	**Sequences (5'–3')**	**Region**	**Nucleotide positions (Range)**
224 Forward	GCIATGYTIGGIACICAYRT	VP1	1977–1996
222 Reverse	CICCIGGIGGIAYRWACAT	VP1	2969–2951
89 Forward	CCAGCACTGACAGCAGYNGARAYNGG	VP1	2602-2627
88 Reverse	TACTGGACCACCTGGNGGNAYRWACAT	VP1	2977-2951
12 Forward	ATGTAYGTICCICCIGGIGG	VP2	2917-2936
22 Forward	GCICCIGAYTGITGICCRAA	VP2	3408-3389
32 Reverse	GTYTGCCA	VP2	3009-3002
